# Supplementary material for: Sulfur-containing class of broad-spectrum antivirals improves influenza virus vaccine development
Source: Nat Commun. 2026 Jan 6;17:1030. doi: 10.1038/s41467-025-67775-5 (PMC12847912; doi:10.1038/s41467-025-67775-5)
Supplement: Supplementary file 1 — Supplementary Information [file 41467_2025_67775_MOESM1_ESM.pdf]

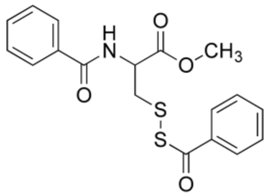

XM-01

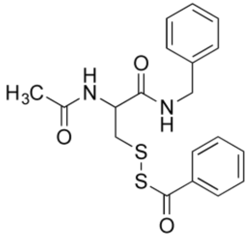

XM-02

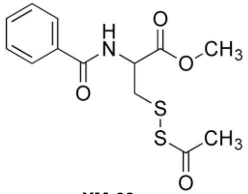

XM-03

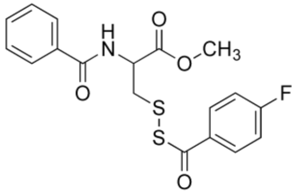

XM-04

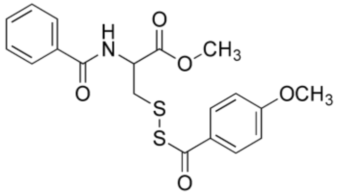

XM-05

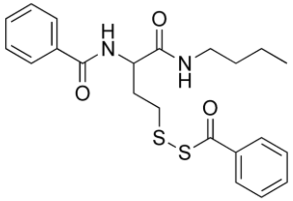

XM-06

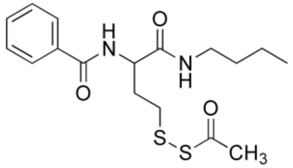

XM-07

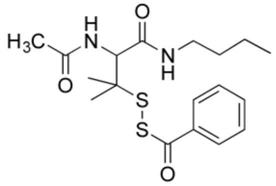

XM-08

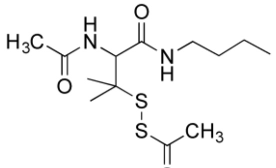

XM-09

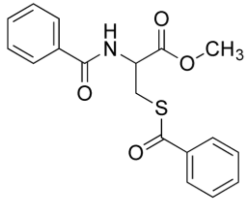

XM-10

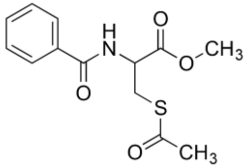

XM-11

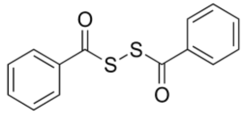

XM-12

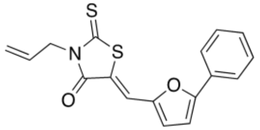

LJ001

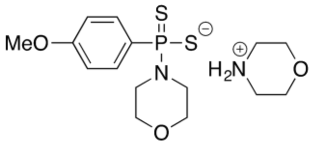

GYY4137

Supplementary Figure 1. Structures of compounds tested (XM- numbers 01-12) and control compounds LJ001 and GYY4137, known to inhibit enveloped viruses.

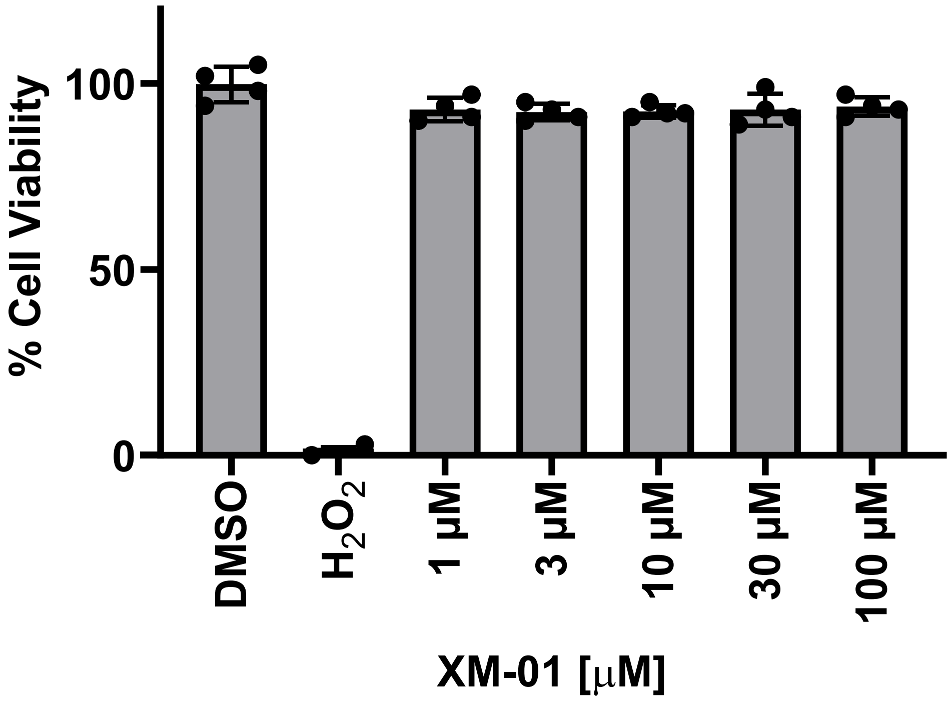

Supplementary Figure 2. XM-01 effect on cell viability was tested from 1 μM – 100 μM in MDCK cells compared to 0.1% DMSO and 2mM H_2O_2 controls. N = 4 independent experiments.

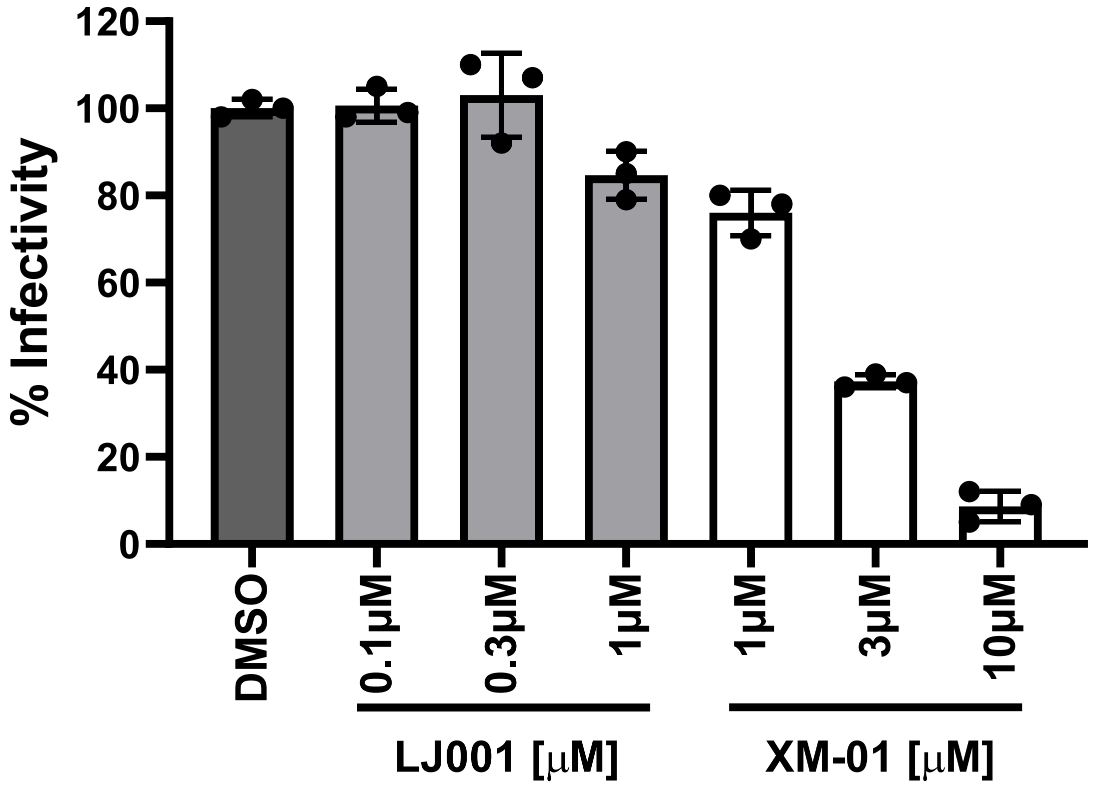

Supplementary Figure 3. Infection of Vero cells by pNiV pre-treated with XM-01 (white bars) and control compound LJ001 (gray bars) before infecting Vero cells in the dark. N = 3 independent experiments.

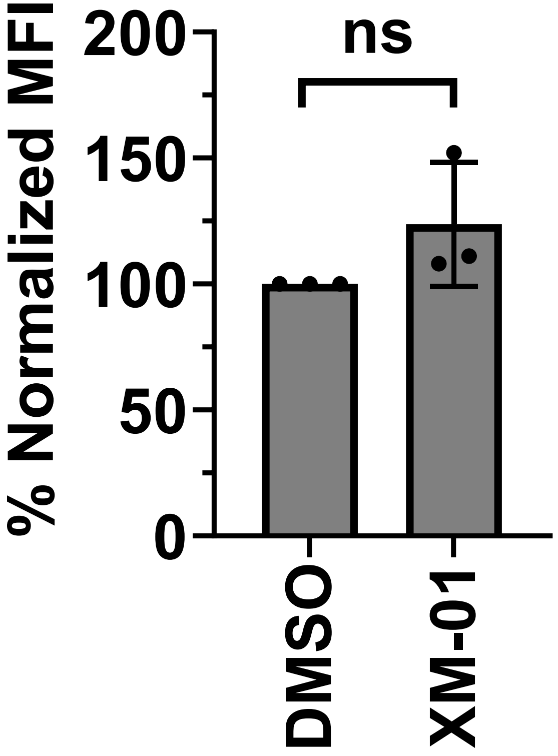

Supplementary Figure 4. Effect of 0.1% DMSO and 10 μM XM-01 treatment of cells expressing NiV-F on triggering in the fusion cascade. Statistical analyses were performed with a t-test. N = 3 independent experiments.

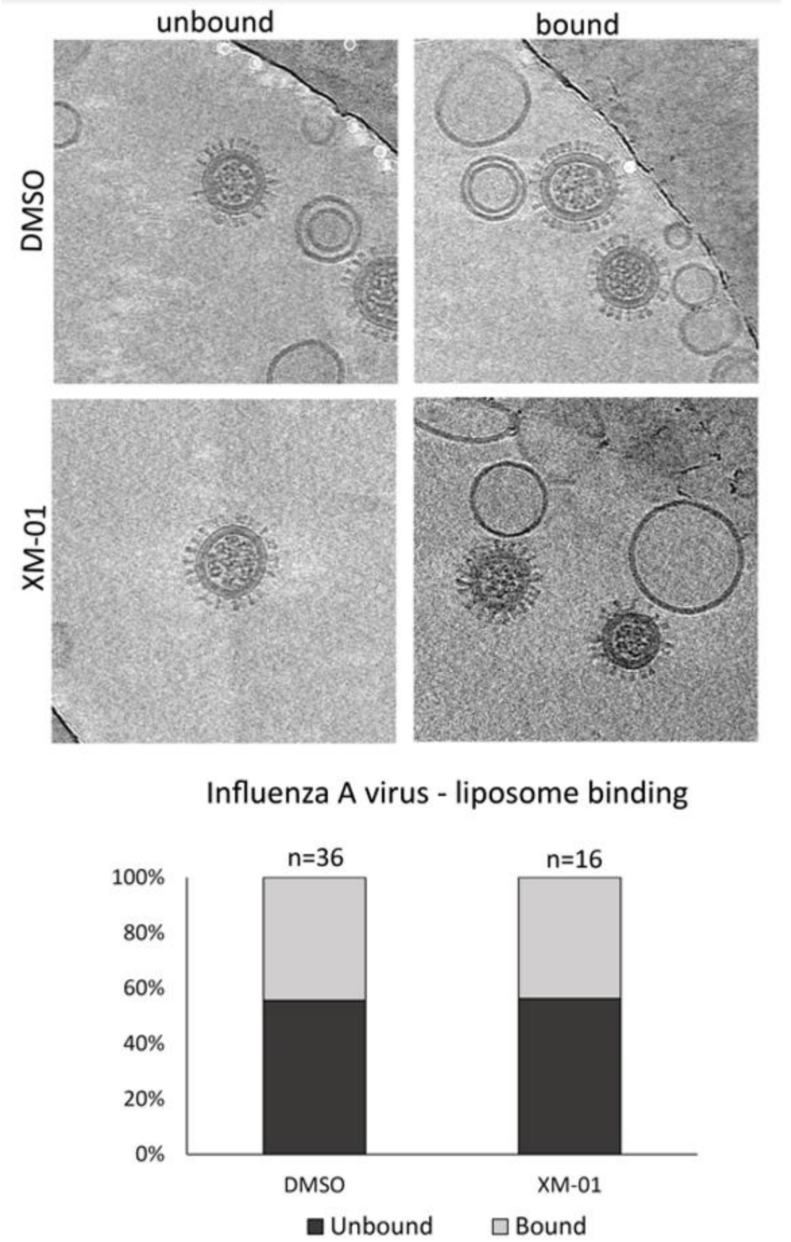

Supplementary Figure 5. Binding of Influenza virus to POPC/cholesterol/total gangliosides (55:40:5, mol/mol) liposomes following treatment with 1 mM XM-01 or DMSO control visualized by cryoelectron tomography.

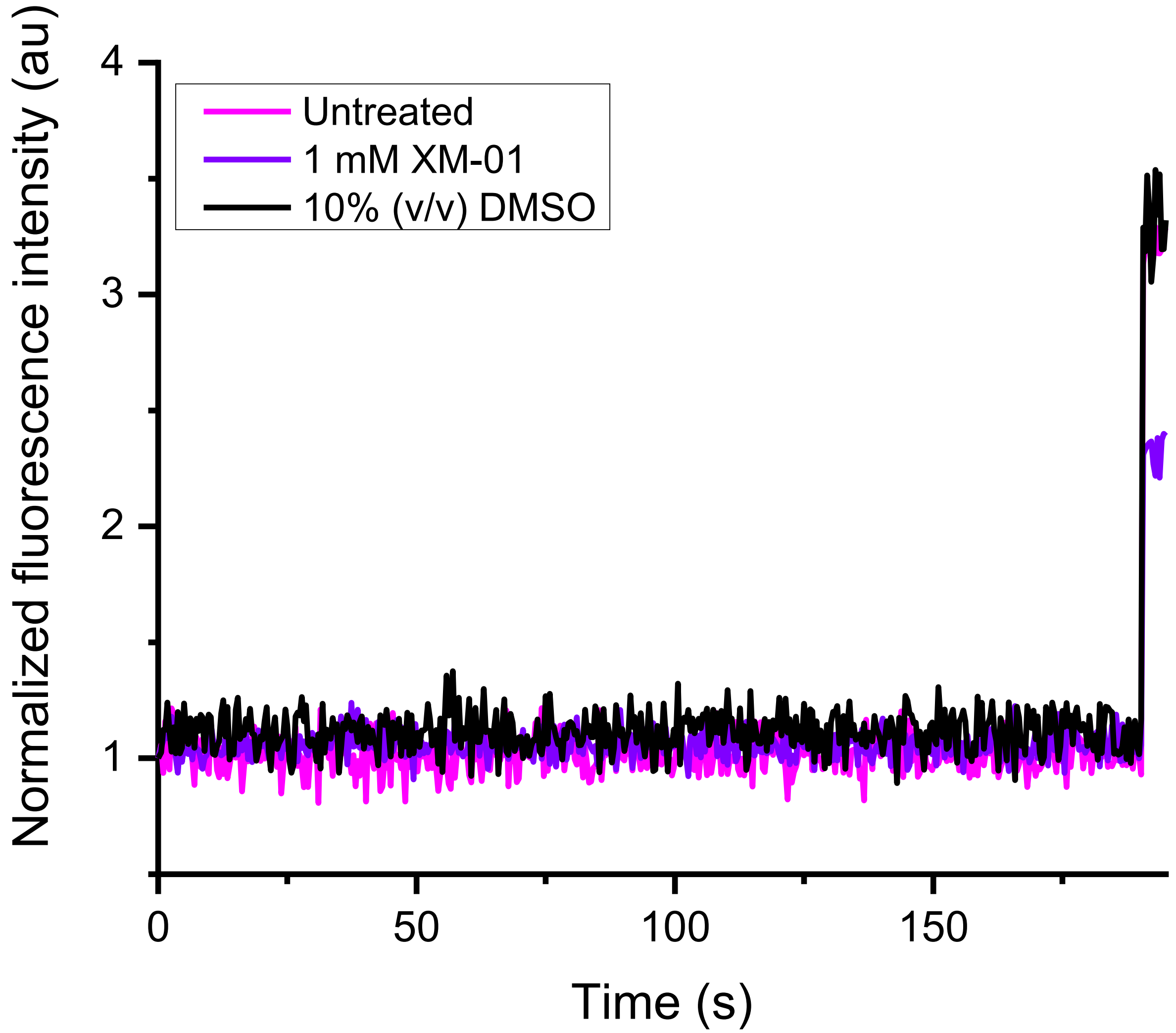

Supplementary Figure 6. Time-lapse of fluorescence intensity measured in untreated, XM-01 treated, and DMSO (vehicle)-treated influenza virus aliquots labeled with the soluble fluorophore sulforhodamine B (SRB) at a semi-quenched concentration regime. The sharp increase in fluorescence intensity beyond t=190 s is due to the addition of a detergent (10% Triton X-100) that leads to release and complete dequenching of the encapsulated SRB molecules. Fluorescence intensity was measured at excitation and emission wavelengths of 565 and 586 nm, respectively, and normalized with respect to fluorescence intensity at t=0 s.

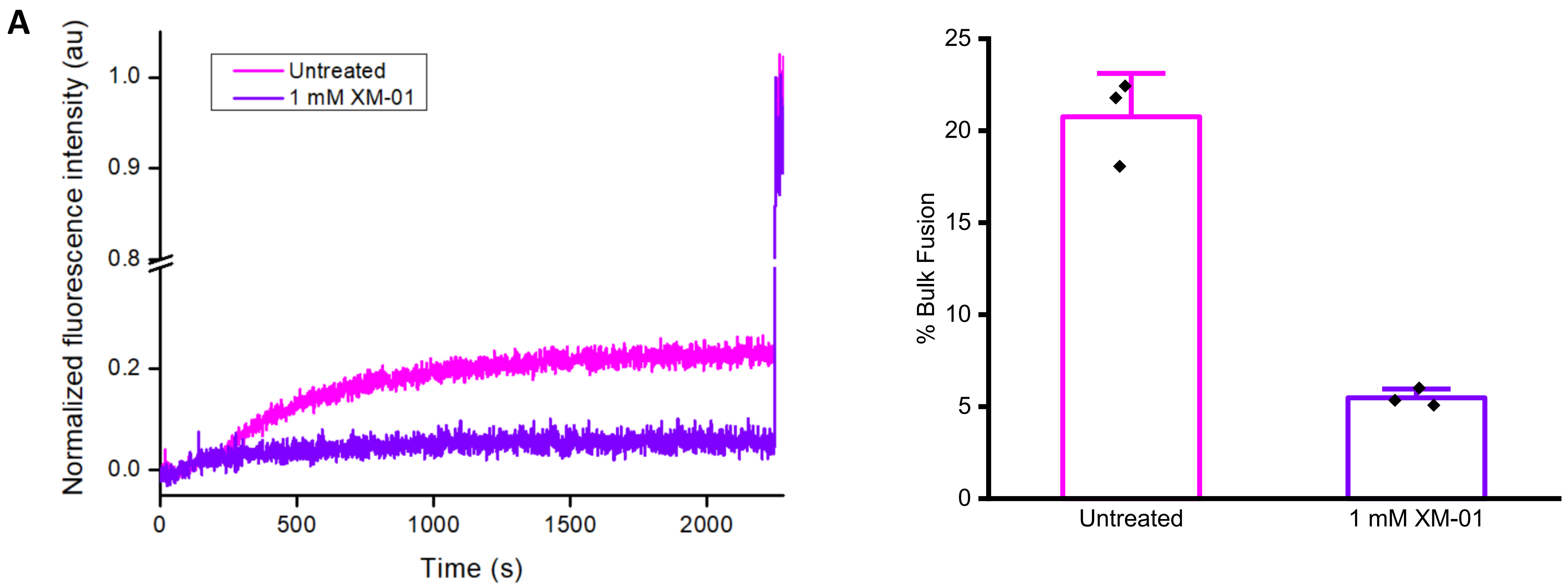

Supplementary Figure 7. XM-01 inhibits bulk fusion of IV with host cell mimicking PMVs. a, Representative bulk fusion traces showing increase in fluorescence intensity of the lipophilic dye R18. **b**, Bar plots showing decreased bulk fusion in the presence of XM-01, relative to that in untreated virus. Increase in fluorescence intensity normalized to the maximum increase in fluorescence intensity on detergent-mediated permeabilization of the viral membrane is plotted on the y-axis as % bulk fusion. N=3

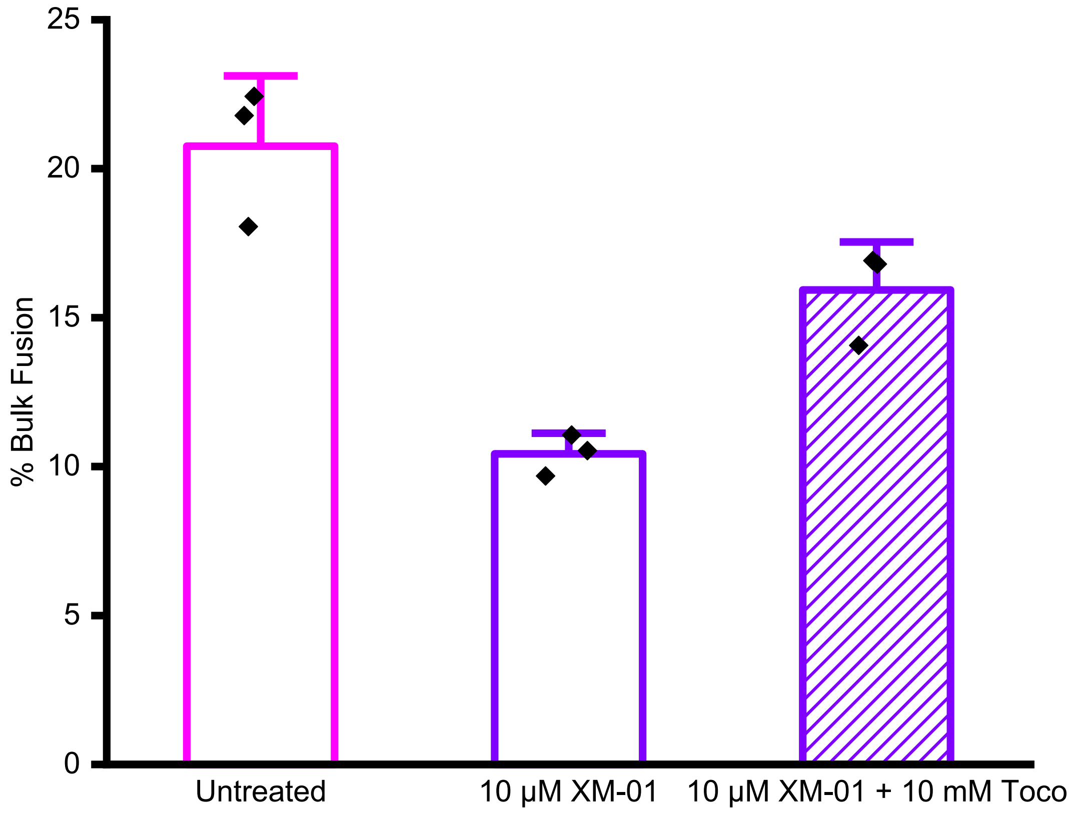

Supplementary Figure 8. **Inhibitory effect of XM-01 on IV fusion is reversed in the presence of free radical quenchers.** Reversal of the effect of XM-01 on IV fusion with host cell mimicking PMVs in the presence of the free radical quencher α-tocopherol, as observed from increase in fluorescence intensity of the lipophilic dye R18. This increase in fluorescence intensity normalized to the maximum increase in fluorescence intensity on detergent-mediated permeabilization of the viral membrane is plotted on the y-axis as % bulk fusion. Concentrations of XM-01 and α-tocopherol are 10 µM and 10 mM, respectively. N=3.

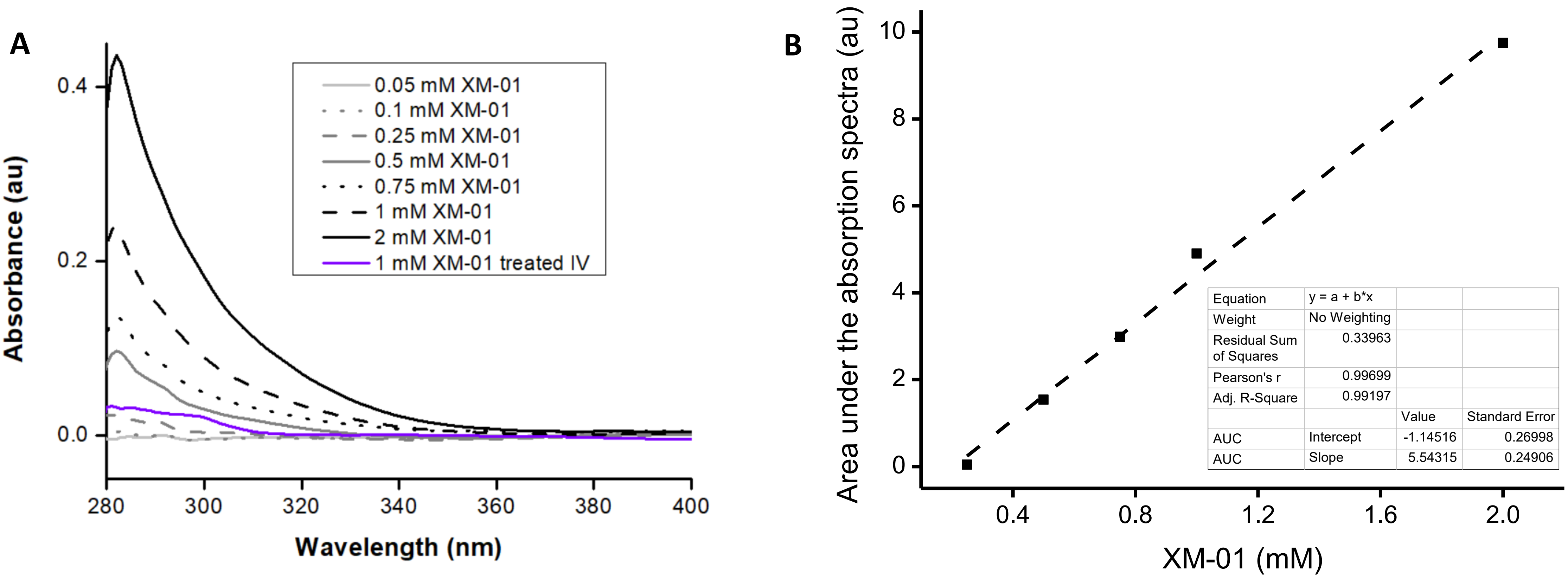

Supplementary Figure 9. Estimation of XM-01 partitioning in IV membranes by absorption spectroscopy. a, Background-subtracted and averaged (N=3) absorption spectra of XM-01 in DMSO and XM-01 partitioned in IV aliquots shown on the left. Spectra for XM-01 samples in DMSO were subtracted from that of DMSO, while spectra of XM-01-treated IV were subtracted from that of DMSO-treated IV. Spectra were moderately smoothened only for the purpose of representation, while ensuring negligible smoothening-induced changes in spectral shape. **b**, Calibration curve showing a linear dependence (adj. R2 value of ~0.99) of area under the background-subtracted absorption spectra for XM-01 in DMSO with concentration.

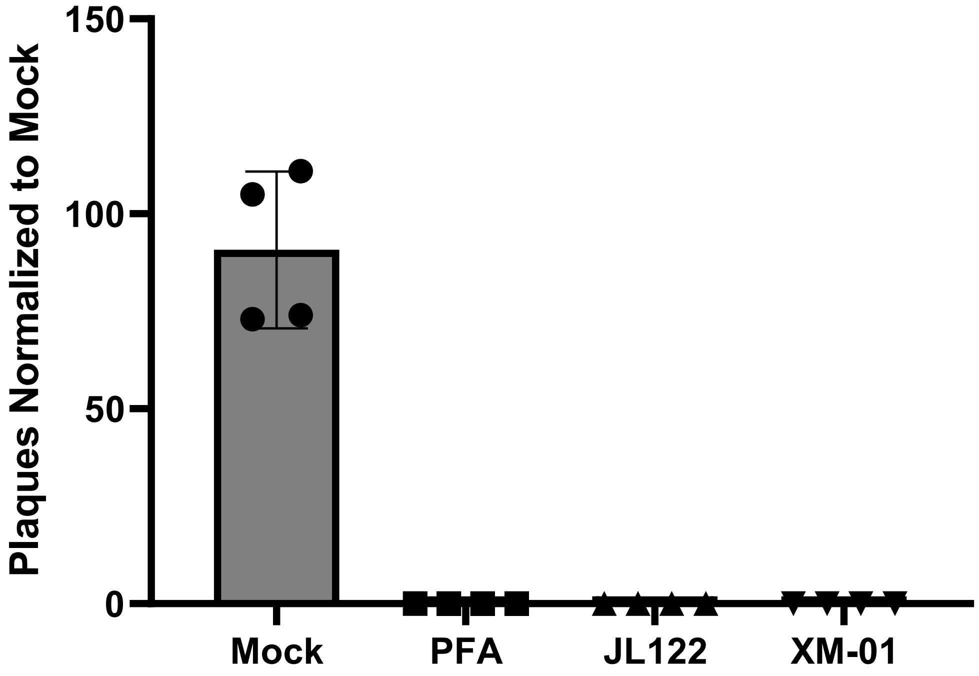

Supplementary Figure 10. Effect of PFA treatment at 0.02%, JL122 at 1uM, and XM-01 at 1 mM on IV determined through plaque assay. N = 4 independent experiments.

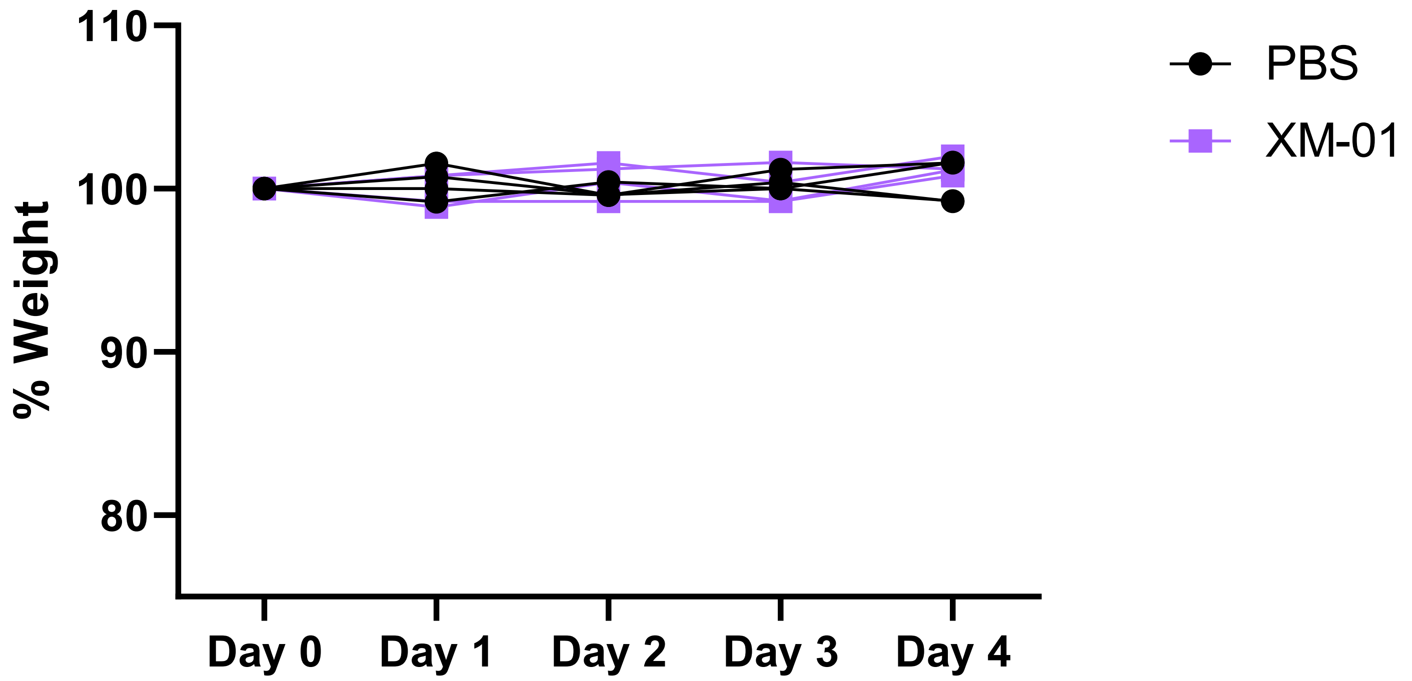

Supplementary Figure 11. XM-01 has no effect on weight loss in mice. Mice received 50 µl of 1 mM XM-01 and weight was monitored for 4 days following IM injection. N=4.

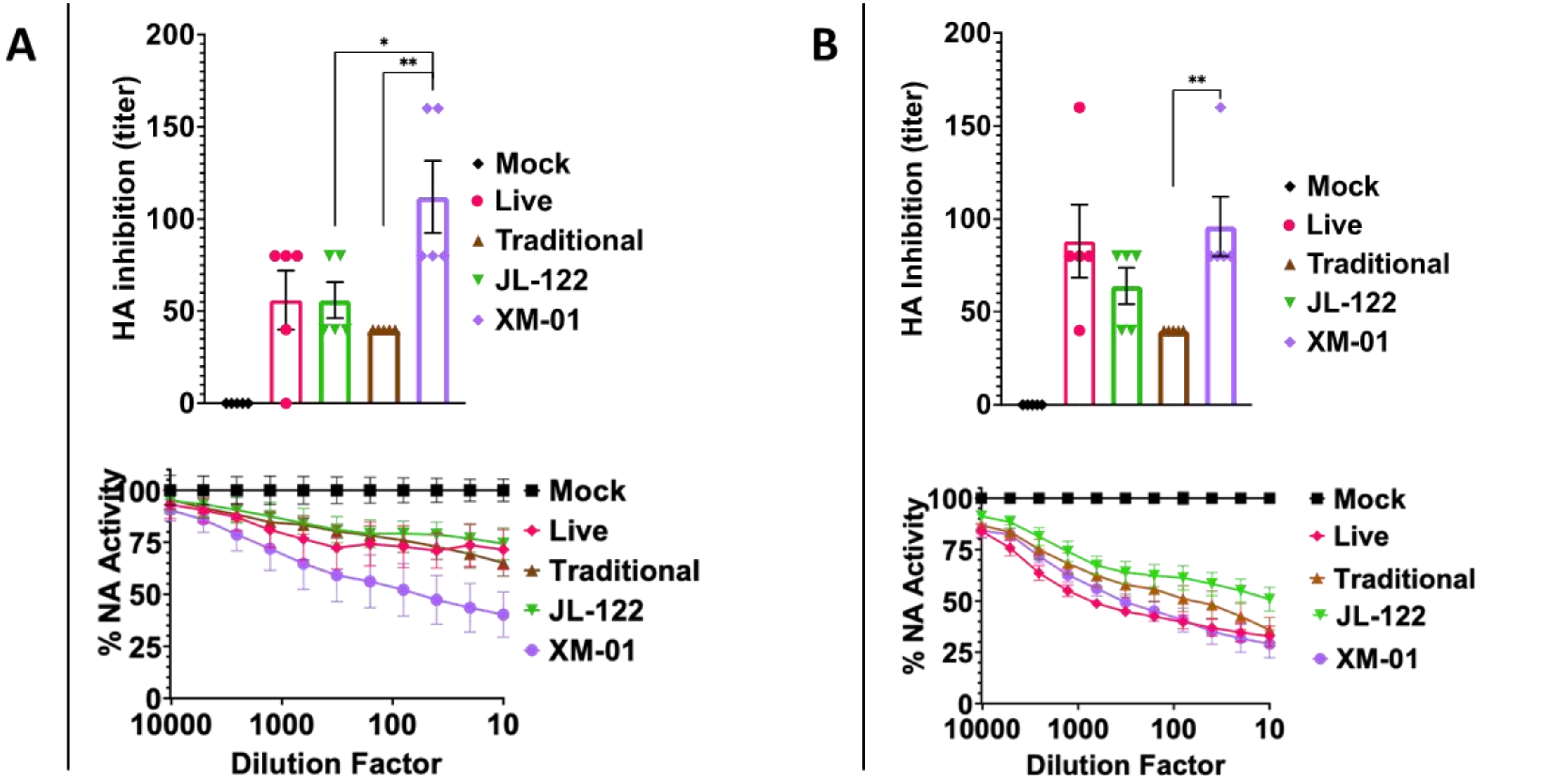

Supplementary Figure 12. Hemagglutination and neuraminidase inhibition assays with serum from vaccinated female mice. a, Female mice serum HA (top) or NA (bottom) inhibition after two vaccinations. **b**, Female mice serum HA (top) or NA (bottom) inhibition after three vaccinations. N = 5 females per group. Statistical analyses were performed using standard t-test (*P < 0.05, **P <0.01).

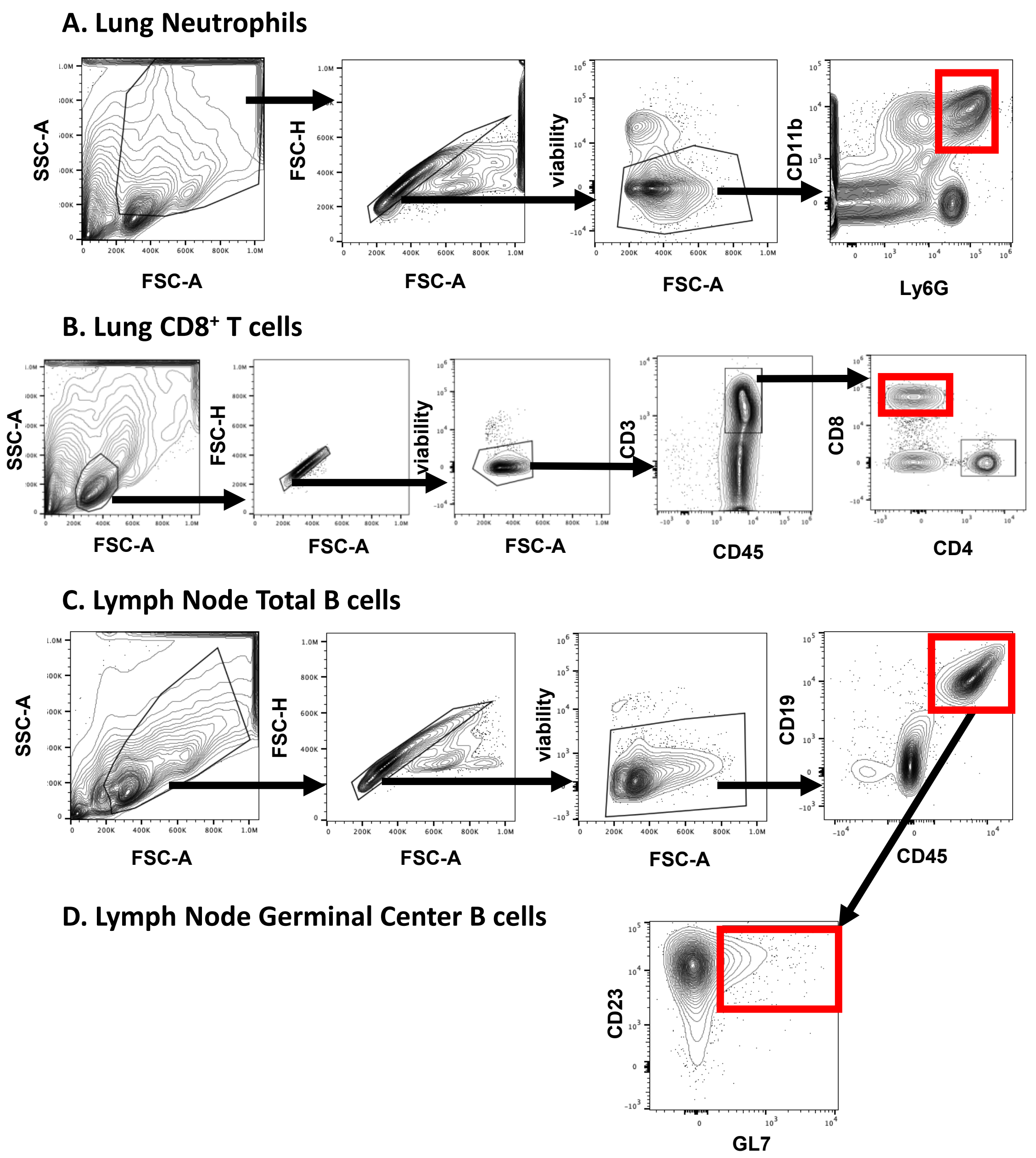

Supplemental Figure 13. Flow Cytometry Gating Strategies for lung neutrophils (**a**), lung CD8+ T cells (**b**), Lymph node total B cells (**c**), or lymph node germinal center B cells (**d**), corresponding to the populations quantified (red boxes) in graphs of Figure 5K-M.
