# Supplementary material for: Sulfur-containing class of broad-spectrum antivirals improves influenza virus vaccine development
Source: Nat Commun. 2026 Jan 6;17:1030. doi: 10.1038/s41467-025-67775-5 (PMC12847912; doi:10.1038/s41467-025-67775-5)
Supplement: Supplementary file 3 — Reporting Summary [file 41467_2025_67775_MOESM3_ESM.pdf]

nature portfolio

Corresponding author(s): Hector Aguilar-Carreno

Last updated by author(s): Dec 03, 2025

Reporting Summary

Nature Portfolio wishes to improve the reproducibility of the work that we publish. This form provides structure for consistency and transparency in reporting. For further information on Nature Portfolio policies, see our Editorial Policies and the Editorial Policy Checklist.

Statistics

For all statistical analyses, confirm that the following items are present in the figure legend, table legend, main text, or Methods section.

n/a	Confirmed	
☐	☒	The exact sample size (*n*) for each experimental group/condition, given as a discrete number and unit of measurement
☐	☒	A statement on whether measurements were taken from distinct samples or whether the same sample was measured repeatedly
☐	☒	The statistical test(s) used AND whether they are one- or two-sided<br>*Only common tests should be described solely by name; describe more complex techniques in the Methods section.*
☐	☒	A description of all covariates tested
☐	☒	A description of any assumptions or corrections, such as tests of normality and adjustment for multiple comparisons
☐	☒	A full description of the statistical parameters including central tendency (e.g. means) or other basic estimates (e.g. regression coefficient) AND variation (e.g. standard deviation) or associated estimates of uncertainty (e.g. confidence intervals)
☐	☒	For null hypothesis testing, the test statistic (e.g. *F*, *t*, *r*) with confidence intervals, effect sizes, degrees of freedom and *P* value noted<br>*Give P values as exact values whenever suitable.*
☒	☐	For Bayesian analysis, information on the choice of priors and Markov chain Monte Carlo settings
☒	☐	For hierarchical and complex designs, identification of the appropriate level for tests and full reporting of outcomes
☒	☐	Estimates of effect sizes (e.g. Cohen's *d*, Pearson's *r*), indicating how they were calculated

Our web collection on statistics for biologists contains articles on many of the points above.

Software and code

Policy information about availability of computer code

Data collection	N/A
Data analysis	N/A

For manuscripts utilizing custom algorithms or software that are central to the research but not yet described in published literature, software must be made available to editors and reviewers. We strongly encourage code deposition in a community repository (e.g. GitHub). See the Nature Portfolio guidelines for submitting code & software for further information.

Data

Policy information about availability of data

All manuscripts must include a data availability statement. This statement should provide the following information, where applicable:

- Accession codes, unique identifiers, or web links for publicly available datasets
- A description of any restrictions on data availability
- For clinical datasets or third party data, please ensure that the statement adheres to our policy

Source data are provided with this paper.

Research involving human participants, their data, or biological material

Policy information about studies with human participants or human data. See also policy information about sex, gender (identity/presentation), and sexual orientation and race, ethnicity and racism.

Reporting on sex and gender	N/A
Reporting on race, ethnicity, or other socially relevant groupings	N/A
Population characteristics	N/A
Recruitment	N/A
Ethics oversight	N/A

Note that full information on the approval of the study protocol must also be provided in the manuscript.

Field-specific reporting

Please select the one below that is the best fit for your research. If you are not sure, read the appropriate sections before making your selection.

[x] Life sciences [ ] Behavioural & social sciences [ ] Ecological, evolutionary & environmental sciences

For a reference copy of the document with all sections, see nature.com/documents/nr-reporting-summary-flat.pdf

Life sciences study design

All studies must disclose on these points even when the disclosure is negative.

Sample size	Sample size was determined based on effect sizes and variability observed in prior influenza vaccination/challenge experiments in our group and similar published work, using group sizes (n = 3-5 animals per group; n = 3 or more independent experiments for in vitro assays) that have consistently provided sufficient statistical power.
Data exclusions	No data was excluded.
Replication	A minimum of 3 biological replicates were used.
Randomization	Mice were allocated into groups randomly.
Blinding	Investigators were blinded.

Reporting for specific materials, systems and methods

We require information from authors about some types of materials, experimental systems and methods used in many studies. Here, indicate whether each material, system or method listed is relevant to your study. If you are not sure if a list item applies to your research, read the appropriate section before selecting a response.

Materials & experimental systems

n/a	Involved in the study	
[ ]	[x]	Antibodies
[ ]	[x]	Eukaryotic cell lines
[x]	[ ]	Palaeontology and archaeology
[ ]	[x]	Animals and other organisms
[x]	[ ]	Clinical data
[x]	[ ]	Dual use research of concern
[x]	[ ]	Plants

Methods

n/a	Involved in the study	
[x]	[ ]	ChIP-seq
[ ]	[x]	Flow cytometry
[x]	[ ]	MRI-based neuroimaging

Antibodies

Antibodies used	Antibodies against mouse antigens included CD16/32 (93; 14-0161-85) Fc block, CD3 (145-2C11; 45-0031-82), CD45 (30-F11; 56-0451-83), CD8 (53-6.7; 11-0081-82), CD19 (eBio1D3(1D3); 11-0193-82), CD23 (B3B4; 25-0232-82), GL-7 (GL-7(GL7); 48-5902-82) from Thermo Fisher Scientific. Ly6G (1A8; 565964), CD11b (M1/70; 563015) from BD Biosciences.<br>We used conformational anti-NiV F and/or anti-NiV G specific rabbit primary antibodies (Anti NiV F Ab 66, or anti-NiV G Ab 213) at 1:100 dilution and secondary Alexa 647 goat anti-rabbit antibodies (Life Technologies, NY).

Validation	These antibodies were validated by providing companies, as well as independently validated in our lab for flow cytometry.

Eukaryotic cell lines

Policy information about cell lines and Sex and Gender in Research

Cell line source(s)	ATCC
Authentication	Original stock was purchased from and validated by ATCC
Mycoplasma contamination	All cell lines tested negative for mycoplasma
Commonly misidentified lines (See ICLAC register)	N/A

Animals and other research organisms

Policy information about studies involving animals; ARRIVE guidelines recommended for reporting animal research, and Sex and Gender in Research

Laboratory animals	8-12 week old C57BL6/J mice were used
Wild animals	N/A
Reporting on sex	Both sexes were tested. Results are shown both as collective and individual sexes.
Field-collected samples	N/A
Ethics oversight	IACUC oversight and guidance.

Note that full information on the approval of the study protocol must also be provided in the manuscript.

Plants

Seed stocks	N/A
Novel plant genotypes	N/A
Authentication	N/A

Flow Cytometry

Plots

Confirm that:

- [x] The axis labels state the marker and fluorochrome used (e.g. CD4-FITC).
- [x] The axis scales are clearly visible. Include numbers along axes only for bottom left plot of group (a 'group' is an analysis of identical markers).
- [x] All plots are contour plots with outliers or pseudocolor plots.
- [x] A numerical value for number of cells or percentage (with statistics) is provided.

Methodology

Sample preparation	Detection of protein conformations by flow virometry. Pseudotyped NiV (pNiV) virions were incubated for 30 min with XM-01 at 4 °C, then washed by ultracentrifugation with NTE buffer (150 mM NaCl, 40 mM Tris-HCl at pH 7.5, and 1 mM EDTA) at 110,000 x RCF for 2 h. The treated virus was resuspended in NTE buffer, then stained as previously described [69]. We used conformational anti-NiV F and/or anti-NiV G specific rabbit primary antibodies (Anti NiV F Ab 66, or anti-NiV G Ab 213) [29, 32, 33] at 1:100 dilution for 1 h, followed by a FACS buffer (1% FBS in PBS) wash and incubation with secondary Alexa 647 goat anti-rabbit antibodies (Life Technologies, NY) for 30 min followed by one more FACS buffer wash. We then measured the relative levels of antibody binding through flow virometry, using a Guava easyCyte8HT flow cytometer (EMD Millipore, MA) [69]. Background mean fluorescence intensity (MFI) was obtained by binding equal concentrations of primary and secondary reagents to mock pseudotyped VSV virus, then subtracted from the MFI of pseudotyped NiV/VSV virions.

Flow cytometry. Lymphocytes were extracted from lung, mediastinal lymph node, and spleen, as previously described [77]. Cells were stained in 30 µL of 1:200 antibodies in PBS for 20 min at RT, washed, and then resuspended for flow cytometry. Fluorescent antibodies used for the study are listed as target (clone; catalog #). Antibodies against mouse antigens included CD16/32 (93; 14-0161-85) Fc block, CD3 (145-2C11; 45-0031-82), CD45 (30-F11; 56-0451-83), CD8 (53-6.7; 11-0081-82), CD19 (eBio1D3(1D3); 11-0193-82), CD23 (B3B4; 25-0232-82), GL-7 (GL-7(GL7); 48-5902-82) from Thermo Fisher Scientific. Ly6G (1A8; 565964), CD11b (M1/70; 563015) from BD Biosciences. Additionally, fixable viability dye (65-0866-14) was used to differentiate live/dead cells. Cells were analyzed on the BD Biosciences FACSymphony, and analyzed with FlowJo Software (BD). Gating strategies are outlined in Supplemental Figure 13.

Instrument

Guave easyCyte8HT and BD Biosciences FACSymphony

Software

FlowJo

Cell population abundance

For all sorting experiments, an aliquot of each post-sort fraction was re-analysed by flow cytometry using the same FSC/SSC, singlet, viability, and marker gates described above. Purity was defined as the proportion of events falling within the predefined “positive” gate for the target marker(s), set using unstained and control-stained samples. In all cases, the sorted fractions were clearly enriched for the desired population relative to the pre-sort sample; the exact post-sort purities for each experiment are reported in the corresponding figure legends.

Gating strategy

For all flow cytometry experiments, cells were first gated on forward scatter (FSC-A) versus side scatter (SSC-A) to exclude debris and to identify the main cell population based on size and granularity. Doublets were removed by sequential gating on FSC-A versus FSC-H (and SSC-A versus SSC-H where applicable) to select singlets. A viability dye was used to exclude dead cells, and all subsequent analyses were performed on live, singlet events.Within this live, singlet gate, cell subsets were identified based on surface marker expression using two-dimensional plots (e.g. Marker 1 vs Marker 2) as indicated in the figure panels. Boundaries between “positive” and “negative” staining populations were defined using unstained and single-stained controls, as well as fluorescence-minus-one (FMO) controls where appropriate, and were kept constant across samples within each experiment. Compensation was calculated using single-stained controls and applied before gating.

☒ Tick this box to confirm that a figure exemplifying the gating strategy is provided in the Supplementary Information.
